# Is optical imaging spectroscopy a viable measurement technique for the investigation of the negative BOLD phenomenon? A concurrent optical imaging spectroscopy and *f*MRI study at high field (7 T)

**DOI:** 10.1016/j.neuroimage.2012.03.015

**Published:** 2012-05-15

**Authors:** Aneurin J. Kennerley, John E. Mayhew, Luke Boorman, Ying Zheng, Jason Berwick

**Affiliations:** Centre for Signal Processing in Neuroimaging and Systems Neuroscience (SPiNSN), Department of Psychology, University of Sheffield, Western Bank, Sheffield S10 2TN, UK

**Keywords:** fMRI, Optical imaging, Haemodynamics, Negative BOLD, Biophysical modelling

## Abstract

Traditionally functional magnetic resonance imaging (*f*MRI) has been used to map activity in the human brain by measuring increases in the Blood Oxygenation Level Dependent (BOLD) signal. Often accompanying positive BOLD *f*MRI signal changes are sustained negative signal changes. Previous studies investigating the neurovascular coupling mechanisms of the negative BOLD phenomenon have used concurrent 2D-optical imaging spectroscopy (2D-OIS) and electrophysiology (Boorman et al., 2010). These experiments suggested that the negative BOLD signal in response to whisker stimulation was a result of an increase in deoxy-haemoglobin and reduced multi-unit activity in the deep cortical layers. However, Boorman et al. (2010) did not measure the BOLD and haemodynamic response concurrently and so could not quantitatively compare either the spatial maps or the 2D-OIS and fMRI time series directly. Furthermore their study utilised a homogeneous tissue model in which is predominantly sensitive to haemodynamic changes in more superficial layers.

Here we test whether the 2D-OIS technique is appropriate for studies of negative BOLD. We used concurrent *f*MRI with 2D-OIS techniques for the investigation of the haemodynamics underlying the negative BOLD at 7 Tesla. We investigated whether optical methods could be used to accurately map and measure the negative BOLD phenomenon by using 2D-OIS haemodynamic data to derive predictions from a biophysical model of BOLD signal changes. We showed that despite the deep cortical origin of the negative BOLD response, if an appropriate heterogeneous tissue model is used in the spectroscopic analysis then 2D-OIS can be used to investigate the negative BOLD phenomenon.

## Introduction

Functional magnetic resonance imaging (*f*MRI) uses localised haemodynamic changes occurring in response to neuronal activity to identify task specific, active areas of the brain. The widely applied Blood Oxygenation Level Dependent (BOLD) *f*MRI signal (Kwong et al., 1992, Ogawa et al., 1992) exploits the paramagnetic properties of deoxy-haemoglobin (Pauling and Coryell, 1936, Weisskoff and Kiihne, 1992) as a marker of neuronal activity. Localised changes in blood oxygenation (Y) resulting from a mismatch of changes in cerebral blood flow (CBF), volume (CBV) and the metabolic rate of oxygen consumption (CMRO_2_) (Buxton et al., 1998) generate a positive BOLD signal change. Often accompanying the positive BOLD signal change are sustained negative signal changes (Alonso Bde et al., 2008, Harel et al., 2002, Kastrup et al., 2008, Pasley et al., 2007, Shmuel et al., 2002, 2006, Smith et al., 2004). The negative BOLD signal is usually, but not exclusively found in cortical regions adjacent to areas of positive BOLD signal change (Smith et al., 2004). Boorman et al. (2010) found (in a rodent model) the negative BOLD signal occurred in deeper cortical layers (1–2 mm) than the positive BOLD signal (0–1 mm). It is uncertain whether this is true generally or specific to their rodent model and/or stimulation paradigm. In the same study concurrent electrophysiology and 2D-optical imaging spectroscopy (2D-OIS) was used in separate subjects to investigate the underlying neuronal activity and haemodynamics respectively. These experiments suggested that the negative BOLD signal was a result of an increase in deoxy-haemoglobin and reduced multi-unit activity in the deep cortical layers. However, Boorman et al. (2010) did not measure the BOLD and haemodynamic response concurrently and so could not quantitatively compare either the spatial maps or the 2D-OIS and fMRI time series directly. Furthermore the study utilised a homogeneous tissue model in which is predominantly sensitive to haemodynamic changes in more superficial layers (< 1 mm) of cortex (Grinvald et al., 1991, 2001, Mayhew et al., 1999). In the study reported below, we use a heterogeneous tissue model (Kennerley et al., 2009) which has greater sensitivity in deeper cortical layers and use concurrent 2D-OIS and 7 Tesla fMRI.

2D-OIS in the visible wavelengths offers a measurement of changes in total haemoglobin (HbT), akin to CBV changes (Kennerley et al., 2005), and blood oxygenation. The benefits of the technique are twofold; data can be recorded with high temporal and spatial resolution; it offers a much cheaper option than *f*MRI for the investigation of the haemodynamic response in the animal model particularly when combined with multi-channel electrophysiology (which can be difficult with *f*MRI). It is therefore important to test whether this measurement technique can used to ‘map’ all aspects of neuronal activity, including the haemodynamics underling negative BOLD signal changes which appear to be biased towards the deeper cortical layers.

To test whether the 2D-OIS technique is appropriate for studies of negative BOLD the current study used concurrent *f*MRI with 2D-OIS for the investigation of the haemodynamics underlying the negative BOLD signal. We extend previously developed concurrent *f*MRI and 2D-OIS methods (Kennerley et al., 2005) for use at high magnetic field strengths (7 T). The increased ^1^H polarisation and signal to noise ratio (SNR) of this higher magnetic field strength allows for more reliable measurements of small negative BOLD signal changes. We investigated whether optical methods can be used to accurately map and measure the negative BOLD phenomenon by using 2D-OIS haemodynamic data to derive predictions from a biophysical model of BOLD signal changes.

We used spatial and temporal 2D-OIS data as input into Monte Carlo Simulations (MCS) of MR signal attenuation (Boxerman et al., 1995) to predict concurrent positive and negative BOLD signal measurements. It has been shown that such biophysical models can be used to predict the superficial 3 Tesla positive BOLD signal change (Martindale et al., 2008). In the current study we tested whether the model holds across field strength by prediction of the 7 Tesla positive BOLD signal from the 2D-OIS data. This justified the use of the MCS of MR signal attenuation model to evaluate whether 2D-OIS measurements have the depth sensitivity to accurately measure the haemodynamic response to study and investigate the negative BOLD signal by comparing MR model prediction to the measured negative BOLD signal changes. We also compared the predictions derived from 2D-OIS data analysed with either a heterogeneous and homogeneous tissue model (parameterised with both functional and structural MRI data — Kennerley et al., 2009). Note that the same baseline blood volume and oxygenation parameters were used in both the MCS of MR signal attenuation and OIS tissue models, for both positive and negative signal changes.

The results showed that 2D-OIS data, when analysed using a heterogeneous tissue model, could be used to investigate the negative BOLD phenomenon. In contrast, analysis using a typical homogeneous tissue model generated predictions which significantly under-estimated the magnitude of both the positive and negative BOLD signal changes.

## Material and methods

### Animal surgery

All aspects of these methods and their development were performed with UK Home Office approval under the Animals (Scientific Procedures) Act 1986. Female hooded Lister rats (total n = 10) weighing (250–400 g) were kept in a 12 h dark/light cycle, at a constant temperature of 22 °C, with food and water ad libitum. Prior to surgery, animals were anesthetised with urethane (1.25 g/kg i.p) with additional doses of 0.1 ml administered as necessary. A single sub cutaneous injection of Atropine (0.4 mg/kg) reduced mucous secretions throughout the experiment. Rectal temperature was maintained at 37 °C throughout surgical and experimental procedures using a homeothermic blanket (Harvard Apparatus Inc, USA). Animals were tracheotomised to allow artificial ventilation. Ventilation parameters were adjusted to maintain blood gas measurements (taken periodically throughout the experiment) within physiological limits (pO_2_ = 105 mm Hg ± 4; pCO_2_ = 38 mm Hg ± 5). The left and right femoral veins and arteries were cannulated facilitating drug infusion and measurement of mean arterial blood pressure (MABP) respectively. Phenylephrine (0.13–0.26 mg/h) was infused to maintain blood pressure between physiological limits (MABP, 100–110 mm Hg) (Golanov et al., 1994, Nakai and Maeda, 1999). Animals were placed in a stereotaxic frame (Kopf Instruments, USA). The skull overlying the right somatosensory cortex was thinned to translucency with a dental drill under constant cooling with saline. An RF surface coil, integrated into a 20 mm diameter Perspex well was fixed to the animals' head using dental cement, ensuring that the thin window lay in the centre of the well.

### Physiological monitoring within the magnet

**Fig. 1:**
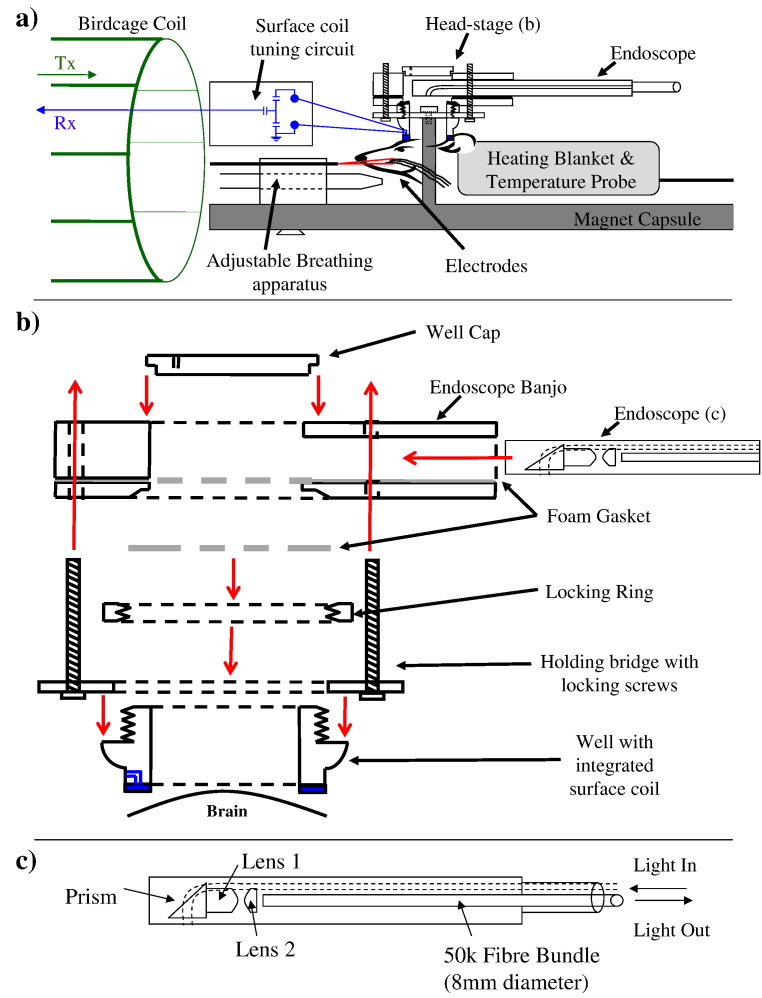
Schematic diagrams of apparatus including a) the magnet capsule containing a heating blanket and temperature probe to maintain the body temperature of the subject whist in the MRI scanner. A large birdcage coil is used for RF transmission. A circular RF receiver coil on top of the subject's cranium is attached to a simple tuning circuit consisting of three non-magnetic variable capacitors. The animal is held still within the magnet using b) a Perspex head-stage. The head-stage consists of the well attached to the subject's cranium (with integrated surface coil). This is screwed onto the holding bridge of the magnet capsule to reduce movement artefacts during the experiment. The endoscope banjo can then be lowered onto the locking screws to position it above the brain. Foam gaskets are used as required because the well is filled with D_2_O to reduce air-tissue susceptibility artefacts and ensure good optical contact for c) the endoscope. The non-magnetic endoscope used for concurrent *f*MRI and 2D-OIS consisting of a 50 K fibre optic bundle and a series of lenses and prisms to allow perpendicular imaging within the small bore 7 Tesla magnet.

Upon completion of the coil attachment procedure the animal was secured within the magnet-compatible holding capsule (Fig. 1a). Inside the capsule an electrically filtered and isolated heating blanket (Harvard Apparatus Inc. USA) and rectal probe, maintained the temperature of the animal. The animal was artificially ventilated (Zoovent Ltd, UK) and blood pressure monitored throughout using a pressure transducer attached to the arterial cannulae (CWE systems Inc. USA). A pressure sensitive pad was placed under the animal to monitor breathing patterns whilst inside the magnet bore (SAII, USA — Model 1025L Monitoring and Gating System). The surface coil was locked to a holding bridge, using a screw on locking ring, thus suspending the head of the animal in the approximate centre of the holding capsule and thus magnet centre following insertion. A non-magnetic endoscope (see below), inserted into a protective Perspex banjo, was subsequently positioned over the surface coil and held in place with locking screws (Fig. 1b). This formed a well which was filled with Deuterium oxide (D_2_O) having a similar refractive index to saline. This reduced optical specularities from the skull surface for 2D-OIS and air-tissue susceptibility artefacts (around the thinned cranial window) for high field *f*MRI whilst not being excited by the 300 MHz RF pulses and consequently avoiding magnetic resonance. The RF feeder cables for the surface coil were attached to the tuning circuit.

The left whisker pad was stimulated with non-magnetic platinum electrodes, insulated to within 2 mm of the tip, and inserted, in a posterior direction, between whisker rows A/B and C/D, ensuring the whole whisker pad was activated following electrical stimulation.

### Four wavelength 2D optical imaging spectroscopy

**Fig. 2:**
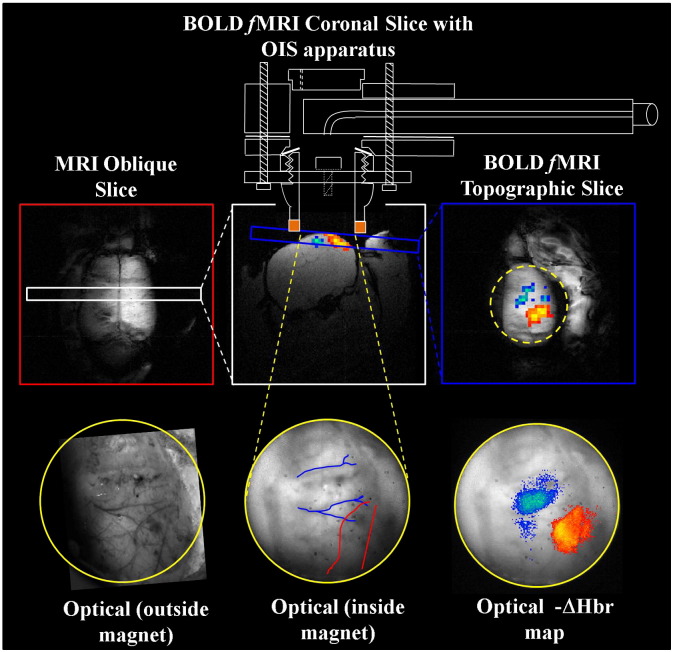
Brain images of experimental orientation. Geometry is given in the anatomical reference frame. An oblique slice covering the dorsal surface of the brain is used to identify a coronal slice containing the whisker barrel cortex (2–3 mm back from the bregma line). This in turn is used to locate a topographic slice over the right hand cerebral hemisphere parallel to optical imaging plane. Apparatus allowing concurrent optical imaging (see Fig. 1) is superimposed onto the coronal MRI section. BOLD *f*MRI statistical activation maps in this topographic plane can be compared directly to complementary functional haemodynamic statistical maps from 2D-OIS. The underlying micro-vasculature can be identified using optical imaging both inside the magnet and outside (without the endoscope). The vessel maps can be used to warp histological sections (see Fig. 3).

Optical imaging spectroscopy (2D-OIS) is a well-established technique for monitoring changes in blood volume (Chance, 1991) and saturation. The imaging technique has found application in the investigation of the underlying mechanisms of the haemodynamic response to increased neuronal activation particularly in animal models (Berwick, 2005, Grinvald et al., 1991, Jones et al., 2001, Malonek and Grinvald, 1996, Malonek et al., 1997, Martin et al., 2002, Mayhew et al., 1999, Nemoto et al., 1997). In the current study 2D-OIS was used to measure changes in the concentration of total, deoxy- and oxy-haemoglobin (HbO_2_, Hbr and HbT respectively). The cortex was illuminated with a white light source built into a switching galvanometer system (Lambda DG-4 Sutter Instruments Company) using 4 wavelength filters (λ — 495 ± 31, 587 ± 9, 559 ± 16 and 575 ± 14 nm). Image data capture within the 310 mm bore of a 7 Tesla magnet (Bruker BioSpecAvance, B/C 70/30 system) used a modified non-magnetic endoscope (Endoscan Ltd, London. Fig. 1c). The 50 K fibre optic bundle had an active diameter of 1 mm and a 7 mm object at a distance of 9.12 mm exactly filled the window. The endoscope tip was a plastic square shaped sleeve ~ 100 mm long so it could be fixed rigidly to the Perspex banjo (Fig. 1b). The active circular window was 6 mm in diameter. This allowed imaging of the whole cranial window and therefore the concurrent technique did not require prior localisation of the area of activation. The endoscope was attached to both the Galvanometer (for light transmission) and a CCD camera (for light reception). The frame rate of the CCD camera was 32 Hz, giving an 8 Hz effective frame rate for each wavelength. The spectrographic data were recorded with a 2D spatial resolution of ~ 80μm * 80μm. Spectral analysis is based upon the path length scaling algorithm (PLSA) described previously (Mayhew et al., 1999) incorporating either a homogeneous or heterogeneous tissue model; the later based on 3 Tesla MRI data (Kennerley et al., 2009). The four wavelength image data set was analysed given the known absorption coefficients for Hbr and HbO_2_ as well as parameterising Monte Carlo simulations of light transport through the two different tissue models with baseline volume and blood oxygen saturation values (Kennerley et al., 2009). This 3D simulation tracks photon transport via a medley of events such as absorption, remittance, scattering and undisturbed propagation to give an estimate of the path length through which the photon has travelled. The distributions of path lengths for a large number of photons were simulated for a range of different tissue parameters (absorption coefficient, scattering coefficient and anisotropy parameter — determining direction change following scattering) over a range of wavelengths. The computed data is entered into a lookup table (LuT). The estimated path-lengths were then used in the Beer–Lambert Law which relates the attenuation of light to the path-length, absorption coefficient and concentration of specific chromophores, to determine changes and produce 2D maps of concentration of HbO_2_, Hbr and HbT (Fig. 2) as a mean across stimuli (see Experimental design).

A baseline oxygen saturation (Y_0_) of 50% was used and assumed to be homogeneous through the cortex. This is in broad agreement with recent studies (Sakadzic et al., 2010) and the classic study by Vovenko (1999) whom both measured the mean oxygen tension pO_2_ for a region of rat parenchyma as ~ 40 mm Hg. The oxy-haemoglobin dissociation curve of blood in rat (Gray and Steadman, 1964) shows that this tension level corresponds to a blood oxygen saturation of ∼ 50%. To further justify this choice of baseline oxygen saturation we investigated the effect, on haemodynamic data, of varying Y_0_ in the spectroscopic analysis between 30 and 90%. Subsequent data were passed into a Monte Carlo of MR signal attenuation to estimate the BOLD signal change at 7 T. Modelled BOLD signal changes peaked in magnitude when Y_0_ was 50%. Varying the Y_0_ over a physiological range (40–70%) had little effect on the BOLD signal magnitude (data not shown). This is in agreement with lower field simulations (Kennerley et al., 2009) and supports the use of 50% as baseline Y_0_.

Baseline blood volume measurements were taken from vessel size index data at 3 T (Kennerley et al., 2005, Tropres et al., 2001). For the homogeneous tissue model we assumed that baseline blood volume fraction was 6%, corresponding to 104 μM. In the heterogeneous model we assumed 6% baseline blood volume fraction in the superficial cortical layers (0–1 mm) dropping to 4% in deeper layers and white matter (1–10 mm).

### Functional magnetic resonance imaging

Once the endoscope allowing 2D-OIS data capture within the magnet core was attached above the cranium the magnet capsule was inserted into the bore of a 7 Tesla magnet (Bruker BioSpecAvance, B/C 70/30 system) with pre-installed 12 channel RT-shim system (B-S30) and fitted with an actively shielded, 200 mm inner diameter, water cooled, 3 coil gradient system (Bruker BioSpin MRI GmbH B-GA20. 200 mT/m maximum strength per axis with 200 μs ramps). A ^1^H quadrature volume resonator (Bruker 1P-T9561, 300 MHz, 1 kW max, outer diameter 200 mm/inner diameter 180 mm) was used for RF transmission and was actively decoupled from the custom built surface coil. A tri-plane fast gradient echo sequence running without phase encoding was used to ensure the surface coil (and thus subject) was positioned at the magnet iso-centre for maximum signal gain. Both resonators were tuned and matched to 300 MHz using the Multlink ^1^H preamplifier with a built-in tune/match display. Once the subject was well localised field shimming, off-resonance correction and RF gains were set and a tri-plane sequence (with phase encoding) was performed for subject localisation. The resulting MR images were used to identify suitable coronal sections of the rat brain for high resolution gradient echo (Fig. 2) scans (256 * 256 pixels, FOV = 30 mm, slice thickness = 2 mm, TR/TE = 1000/15 ms, flip angle = 90°, 2 averages). Further high resolution transverse scans covering the dorsal surface of the brain allowed accurate localisation of the rodent whisker barrel cortex (2 mm back from the visible bregma line — Fig. 2). A single oblique/topographic slice (covering the surface of the right hand cortex and in a similar plane to OIS data — Fig. 2), was performed using the same scan parameters as above.

Functional data were acquired from suitable topographic slices using a single shot MBEST Gradient Echo — Echo Planar Imaging (GE-EPI) sequence during electrical stimulation of the whisker pad (raw data matrix = 64 * 64, data sampling interval = 5 μs, FOV = 30 mm, slice thickness = 2 mm, TR/TE = 1000/12 ms, flip angle 90°, 10 dummy scans). Read-out direction was left–right. Standard phase correction (Bruder et al., 1992) was used to minimise Nyquist ghosting. The BOLD signal was calculated as fractional change normalised by the mean of a one minute preliminary baseline signal (see Experimental design below).

### Experimental design

Haemodynamic changes were induced by electrically stimulating the left whisker pad at an intensity of 1.2 mA, and at a frequency of 5 Hz for 16 s. This stimulation frequency was chosen as it produces the largest haemodynamic response in the somatosensory cortex of anesthetised rodents whilst maintaining stable physiological parameters (Martin et al., 2006). In all experiments an initial baseline of 60 s was collected followed by thirty stimulation events each with an inter stimulus interval (ISI) of 70 s. The BOLD signal was calculated as fractional change normalised by the baseline mean, recorded during the initial 60 s control period. All stimulus and optical imaging timing control were performed using a CED1401 (using a Spike-CED, UK, interface) which was triggered to start using a TTL pulse indicating MR echo acquisition. Optical imaging data was set to be collected for all stimulus trials with a 10 s baseline period and for a duration of 70 s (covering the ISI). During spectral analysis 2D-OIS data is collapsed across trials.

### Data analysis

Both 2D-OIS and *f*MRI data analyses were conducted using Matlab (The MathWorks Inc. USA). After GE-EPI to GE structural image registration all *f*MRI data were statistically analysed using the general linear model (GLM) approach as in SPM (Friston et al., 1991).

The time series across each pixel was compared to a design matrix of a representative boxcar haemodynamic response function, a ramp and a DC offset. Activation z-scores were calculated on a voxel by voxel basis. These scores were then superimposed on detailed structural scans to show active regions. Regions were positively and negatively thresholded to include trials which produced an ‘active’ region greater than five adjacent voxels, with a z-score for each voxel greater than four (for positive BOLD) and less than minus two (for negative BOLD). Time series of responses was taken as the mean across trials and subjects for direct comparison with OIS data. OIS regions of positive and negative changes in Hb0_2_, Hbr and Hbt, were found using the same GLM and method as the MRI data. After taking the mean time series it was unnecessary to further filter the time series data from either imaging modality.

In order to compare 2D-OIS and fMRI spatial maps we adopted the following procedure. 2D-OIS measurement data of changes in Hbt and Hbr were input into the MCS of MR signal attenuation to obtain spatio-temporal predictions of the BOLD data. The spatial BOLD prediction was then analysed using GLM with a box-car response model (as above). These preliminary z-score maps were used to identify approximate regions of positive and negative BOLD containing peak z-score values. Mean time series data from these regions were subsequently used to refine the design matrix. The 2D-OIS BOLD predictions and fMRI data (interpolated to 8 Hz) were analysed using this temporal design model.

The parameter values corresponding to the magnitude of the positive and negative BOLD response were used as spatial map of the predicted BOLD (from 2D-OIS data) and the fMRI BOLD response. The fMRI BOLD map was smoothed using a 2D-Gaussian filter (sigma = 489μm corresponding to the FOV scale voxel). The prediction map was sub-sampled to equivalent spatial resolution and cropped to a region surrounding the active areas to form a template (no further filtering or smoothing other than the cubic interpolation intrinsic to Matlab was used). This template was subsequently spatially correlated with the normalised fMRI parameter map. The template was rotated and translated to find the maximum normalised correlation between the prediction and measurement maps.

### Cytochrome oxidase and photographic emulsion histology

After completion of the experimental paradigms n = 7 rats underwent cytochrome oxidase and photographic emulsion histology. This histological method has been previously described in Zheng et al. (2001) but a brief description is given here. Rats were transcardially perfused with saline followed by a 4% paraformaldehyde and vessels were filled with photographic emulsion (Jessops Ltd.). The right cortex was removed and compressed to a thickness of 2 mm. The cortex was then sectioned on a cryostat. A 200 μm thick slice was taken and included all surface vessels. Subsequent sequential 50 μm slices were taken from this point. Using a modified version of the procedure from Wong-Riley and Welt (1980) the slices were placed in the dark in an incubation medium (37 °C) to stain for cytochrome oxidase. The photographic emulsion contained within vessels was developed post staining.

**Fig. 3:**
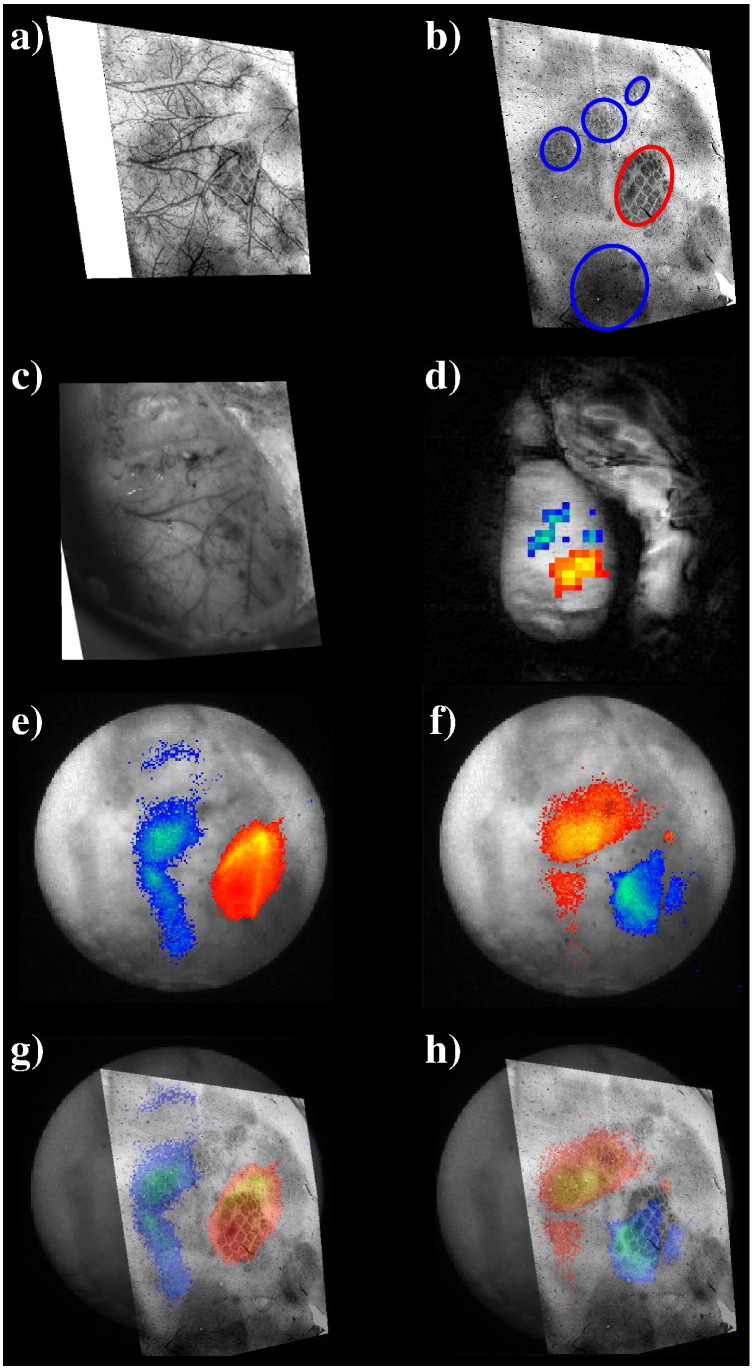
Histology and functional activation maps. a) Post mortem histology showing stained barrel cortex and overlying vasculature. b) The same section with the vasculature removed. c) Live image of cortex outside of magnet allows easy visualisation of vasculature or comparison to images taken inside the magnet through the endoscope. d) BOLD *f*MRI SPM map showing positive BOLD changes in whisker barrel region and negative BOLD changes in surrounding somatosensory cortex in response to electrical stimulation of the whisker pad. e) Map of underlying HbT changes. f) Map of underlying Hbr changes. Using vascular net it is possible to warp the live images to the post mortem histology to overlay activation maps onto barrel stained sections for g) HbT and h) Hbr. Images indicate ‘positive’ BOLD is located in whisker barrels; negative BOLD in surrounding somatosensory cortex e.g. forepaw/hind paw.

Images of the stained slices can be linearly warped to one another where corresponding features are superimposed (Fig. 3a). The mathematical details of the warping have been described previously (Zheng et al., 2001). Penetrating blood vessels are used as fiducial markers between sections. Four matching points in each image defined an exact projection between the points; however, it was preferable to use a larger number of corresponding points to calculate the best (in least squares sense) projective transform. The surface section acts as a base template. The section immediately below is warped to fit over the surface section and then the section containing the barrel cortex (Fig. 3b) is warped to fit over the previously warped second section. The surface and warped barrel images are then superimposed to create a barrel map on the surface of the cortex to compare with the imaging results. For comparison with optical imaging results the surface section containing the stained vasculature is warped (using a similar method to that described above) to the vasculature seen in the endoscope images (Fig. 3c). The same warping parameters can then be applied to the histological section showing the stained somatosensory barrels and haemodynamic z-score maps from 2D-OIS superimposed to assess which cortical regions are active.

## Results

We investigated the signal source of both the positive and negative BOLD *f*MRI signal changes using a biophysical model of BOLD which relates the *f*MRI signal to the underlying haemodynamics (Martindale et al., 2008). We utilised a concurrent *f*MRI and 2D-OIS methodology for data collection.

### Topographic mapping

A representative topographic section BOLD signal statistical parameter map (superimposed onto a 256 * 256 GE-structural image) of the right cortical hemisphere in response to electrical stimulation (1.2 mA, 5 Hz, 16 s) of the left whisker pad is shown in Fig. 3d. We found a statistically robust (z > 4) evoked positive BOLD response confined to the whisker barrel field. Anterior and medial to this response we found a significant (z < − 2) negative BOLD response. The concurrent maps of changes in total (HbT) and deoxygenated haemoglobin (Hbr) are shown in Figs. 3e and f respectively. In the whisker barrel cortex we found increases in HbT and decreases in Hbr. In agreement with previous work (Kennerley et al., 2005), the major increases in HbT were located primarily along the arteries (Lee et al., 2001) whereas decreases in Hbr were located primarily in areas of parenchyma. In areas anterior and medial to the whisker barrels we found decreases in HbT and increases in Hbr, the later predominantly generating the negative BOLD signal. Superimposing haemodynamic maps onto histological sections (Figs. 3g–h) of the cortex showed that the haemodynamics driving the negative BOLD were located in regions corresponding to the forelimb, hind-limb and trunk regions of the somatosensory cortex. Results were similar across all subjects (n = 10) in the present study.

**Fig. 4:**
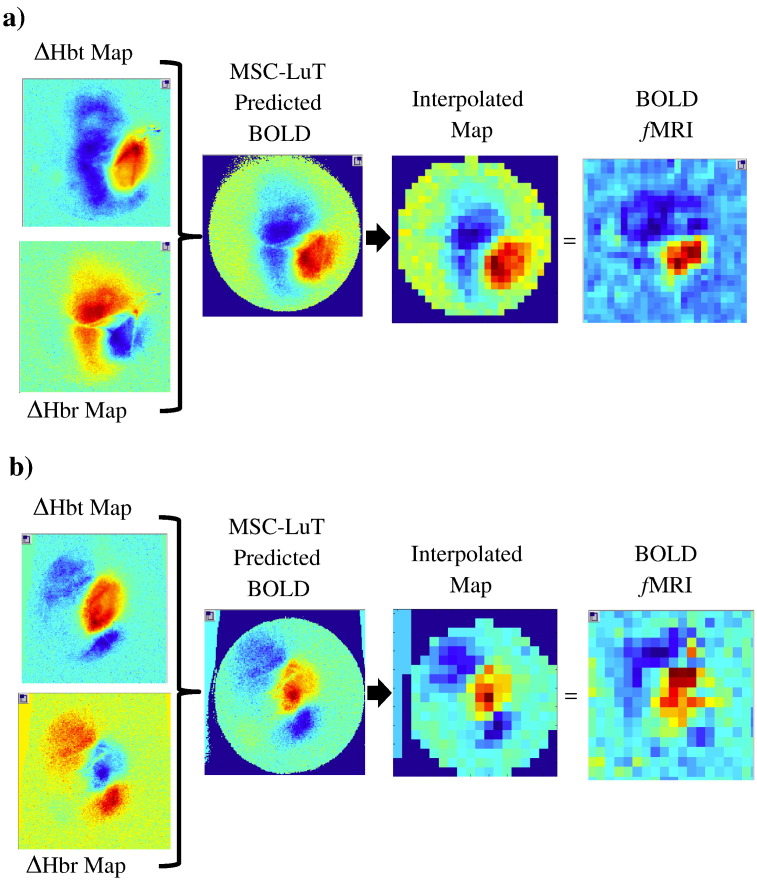
Spatial predictions of BOLD from underlying haemodynamics for 2 representative animals. Spatial maps of changes in HbT and Hbr are input into a Monte Carlo simulation of MR signal attenuation to predict the BOLD signal. The resulting BOLD prediction is subsampled so it can be directly compared to the concurrent *f*MRI data.

The spatial concordance between *f*MRI and 2D-OIS data was assessed by using the spatial 2D haemodynamic data in a Monte Carlo simulation of MR signal attenuation as in Martindale et al. (2008). The predicted BOLD signal was sub-sampled to the same spatial resolution as the *f*MRI BOLD data as described in the Material and methods section (Fig. 4 shows results from 2 representative z-score maps). As can be seen, for both positive and negative BOLD signal changes, there was concordance between measured and predicted activation maps despite differences in measurement resolution and technique.

The resolution of the BOLD fMRI EPI-data was insufficient to resolve the surface vasculature necessary for co-registration to the predicted BOLD maps derived from the 2D-OIS data. We nevertheless investigated the spatial correlation using the method described in the Material and methods section (data analysis). Briefly, a template of the predicted BOLD GLM parameter map was warped with an affine transformation (uniform scale, translation and rotation) to the fMRI BOLD parameter map. For combined parameter maps of both positive and negative BOLD response the mean normalised correlation coefficient was 0.86 (± 0.02). For the positive BOLD response alone the mean normalised correlation coefficient was 0.91 (± 0.01). As expected, as the SNR for the positive BOLD response is higher than the negative BOLD response the negative only correlation was 0.78 (± 0.02).

We can estimate the scaling factor from the inner diameter of the RF coil (which forms the optical well) and the number of pixels spanning this diameter in the 2D-OIS images. The scaling factor to subsample the 2D-OIS data to the same resolution as the fMRI data should be of the order 0.18. The scale determined by the affine transformation to maximise spatial correlation was found to be 0.15. Whether this is due to the intrinsic blurring of 2D-OIS, EPI distortions, our choice of Gaussian smoothing of fMRI map, or more prosaic causes (e.g. dental cement reducing the optical window) is uncertain. On competition of future experiments utilising this concurrent 2D-OIS and fMRI methodology, one can replace the D2O with H2O, image the well directly with EPI sequences to better estimate this scaling factor. Nevertheless it is clear from these results that there is a potentially useful correspondence between 2D-OIS data and BOLD fMRI data.

### Time series data

**Fig. 5:**
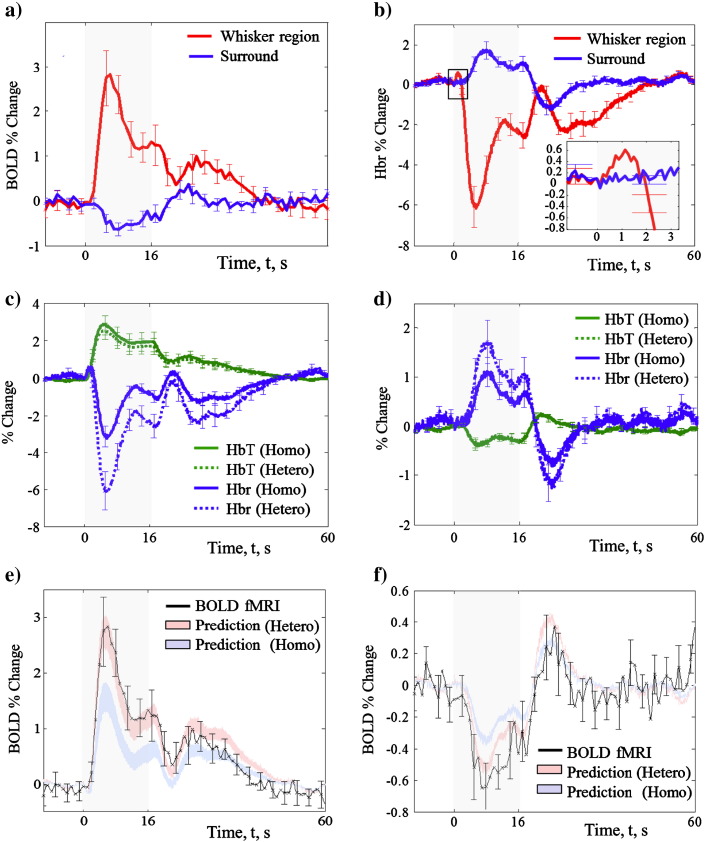
Time series data of a) positive and negative BOLD measured with *f*MRI, b) the concurrent underlying Hbr measurements from both regions. The whisker barrel (positive BOLD) region shows the ‘deoxy dip’ the surrounding cortical (negative BOLD) region does not (insert). 2D-OIS haemodynamic data for c) the whisker barrel/positive BOLD and d) surrounding cortex/negative BOLD region is analysed with both heterogeneous and homogeneous tissue models. The former shows increased magnitude of Hbr changes. Haemodynamic data is input into a MCS of MR signal attenuation to predict e) the positive BOLD and f) the negative BOLD using both hetero- and homogeneous tissue models. Predictions are for the extra-vascular space assuming a mean vessel radius between 3 and 20 μm (generating the prediction envelopes shown). For both regions we find good agreement between *f*MRI measurements and model predictions only when using the heterogeneous tissue model in 2D-OIS spectral analysis.

Region of interest masking of threshold activation maps (whisker and surrounding cortex) was used to obtain time series data from a mixed region of arterioles, parenchyma and venules (Fig. 5). The average time series (mean across all subjects), of the BOLD *f*MRI signal showed a positive signal change in the whisker barrel cortex in response to electrical stimulation of the whisker pad (Fig. 5a). The response peaked with a magnitude of ~ 3.5% at 4 s after stimulus onset, falling to a plateau (~ 1.5%) which was maintained until stimulus offset. In regions surrounding the whisker barrel cortex the time series showed reliable negative BOLD peaking with a magnitude of ~−1% at 8 s (Fig. 5a).

Concurrent measurements of the underlying Hbr changes in both the whisker barrel and surround cortex are shown in Fig. 5b. We found a corresponding decrease and increase of Hbr levels in the whisker barrel and surrounding cortical regions respectively. Each inverted Hbr time series showed statistical Pearson product–moment correlation (group data = 0.97) with the observed BOLD signal changes.

Concurrent haemodynamic time series from spectroscopic analysis of 2D-OIS data using both a heterogeneous and commonly used homogeneous tissue model for light transport through tissue are shown in Figs. 5(c) and (d) for the whisker barrel cortex and surrounding regions respectively. It is clear that the different tissue models used for spectroscopic analysis have little effect on the temporal transients of the resulting haemodynamics (Kennerley et al., 2009); but seriously affect the magnitude of response changes. We show direct evidence that the negative BOLD response is a product of increased Hbr and decreased HbT changes — contrasting with the haemodynamic response driving the positive BOLD signal (in the whisker barrel cortex).

2D-OIS haemodynamic time series data of HbT and Hbr changes were input into a Monte Carlo simulation of MR signal attenuation (Martindale et al., 2008) to estimate corresponding changes in BOLD signal. Both the hetero- and homogenous tissue model predictions were evaluated. BOLD signal estimates derived from the 2D-OIS data were directly compared to the concurrently measured BOLD *f*MRI signal changes in both the whisker barrel cortex (Fig. 5e) and surrounding cortex (Fig. 5f). Predictions were of the extra-vascular space assuming a mean vessel radius between 3 and 20 μm (Pawlik et al., 1981); generating the prediction envelope shown in the figures.

### Tissue model evaluation

For both the whisker barrel and surround cortex we found good agreement (within standard error) between both positive (Fig. 5e) and negative (Fig. 5f) BOLD *f*MRI measurements and model predictions when using the heterogeneous tissue model in 2D-OIS spectral analysis. Both the transients and amplitude of response were captured by the model. In contrast, 2D-OIS analysis using a homogeneous tissue model, although showing similar transients, significantly under-estimated the magnitude of both the positive and negative BOLD *f*MRI signal changes. This data supports the use of more detailed MR pre-parameterised tissue models for the analysis of 2D-OIS data (Strangman et al., 2002).

## Discussion

The present study investigated the haemodynamic response underlying the BOLD signal through utilisation of concurrent 2D-OIS and high field 7 Tesla *f*MRI methods. We observed a robust positive BOLD response in the whisker somatosensory barrel cortex. In surrounding cortical areas we found a reliable negative BOLD signal. We used 2D-OIS haemodynamic measurements in a Monte Carlo simulation of MR signal attenuation in an attempt to model the concurrently measured BOLD *f*MRI signal. When optical imaging data is analysed with an MR parameterised heterogeneous tissue model (Kennerley et al., 2009) can we reliably predict the temporal dynamics and magnitude of the concurrent positive and negative BOLD *f*MRI signal changes in the whisker barrel and surrounding cortex respectively. Despite the negative BOLD response occurring at a greater depth in the cortex than the positive BOLD response (Boorman et al., 2010), data presented here show that optical imaging spectroscopy with an appropriate heterogeneous tissue model can be used to investigate the negative BOLD phenomenon.

The prediction errors were slightly greater for the negative BOLD than the positive BOLD (Figs. 5e and f). This may be due to either i) negative BOLD occurring at the limit of the depth sensitivity for 2D-OIS, ii) ‘weakness’ in the MR MCS or a combination of both.

### Depth sensitivity

Results from Boorman et al. (2010), using the same rodent model, show neuronal activity and resulting negative BOLD peaks at 1.3 mm cortical depth. This is close to the limit of sensitivity of 2D-OIS using the visible wavelengths using homogeneous tissue models. The heterogeneous tissue model was designed to improve estimations of the haemodynamic as a function of cortical depth (Kennerley et al., 2009).

The heterogeneous model (and spectroscopy algorithm) cannot selectively exclude superficial layers, it can only be biased to respond proportionally more to deeper changes. In contrast, if biased towards the superficial layers the resulting data would have reduced contribution from changes in deeper cortical layers. Currently this bias is relatively coarse; splitting the tissue model into only 5 layers (see Kennerley et al., 2009).

Nevertheless, using the prior that neuronal activity driving the negative BOLD is predominantly in the deeper cortical layers we can bias the spectroscopy algorithm to be more sensitive to changes in the deeper layers of the cortex. Fig. 7 shows that the heterogeneous model gives a better prediction of the time series of negative fMRI-BOLD signal. This can be seen from the fact that the heterogeneous model gradient is very close to 1. The correlation of the prediction to measurements is both very high (with different tissue models) which implies that the effect of the heterogeneous tissue model is primarily one of scaling.

Detailed quantification of the depth sensitivity of the heterogeneous tissue model would require improved estimates of the 3D distribution of scattering and absorption across more cortical layers and thus is beyond the scope of this paper and is on-going research. However, Figs. 5(e) and 7 show how the use of parameterised depth sensitivity of tissue parameters for the analysis of spectroscopic data improves the fit between the predicted and measured BOLD signals. Results therefore suggest that 2D-OIS can be used in the investigation of the negative BOLD phenomenon if one includes a layered heterogeneous tissue model in the spectral analysis.

### Refinement and improvement of MCS-MR signal attenuation

At the current *f*MRI single shot EPI resolution (~ 500μm^2^ in plane) our data showed that it is sufficient to use a single homogeneous-voxel (representing parenchyma with a mean vessel radius 3–20μm: Pawlik et al., 1981) MCS of MR signal attenuation to map both the mean positive and negative BOLD from the underlying haemodynamics.

Recent studies using optical coherence tomography with confocal phosphorescence (Yaseen et al., 2011) demonstrate haemodynamic and oxygenation changes in larger vessels (up to 150 μm) than those used as our range in the MCS of MR signal attenuation. However, this range only acts a parameterisation of the tissue vasculature within an fMRI voxel (~ 400 * 400 * 2000 μm in this study) for the MCS. Any large vessels (up to 150um) only form a small fraction of the voxel volume. Thus, this would only increase the ‘mean’ vessel radius towards the higher limit of our applied range. Furthermore, the MCS of Boxerman et al. (1995), repeated by Martindale et al. (2008), clearly demonstrate that at vessel radii above 15 μm there is a little change in the Gradient Echo MR signal. Hence the range applied in this study would be adequate to account for these large vessels if they did form a more significant proportion of the voxel.

With the improved spatial resolution of parallel MR imaging techniques it may be necessary to refine the MCS to include a 3D heterogeneous model of the brain tissue. The improved spatial resolution of parallel imaging may allow investigation of the BOLD signal in different vascular compartments which would require different physiological variables (e.g. baseline blood volume fraction, oxygen saturation, vessel size etc.) and intra and extra vascular (IV/EV) signal proportions. At a field strength of 7 T and for small capillary sized vessels (~ 3 μm radius), where negative BOLD originates, 7% of the BOLD *f*MRI signal is from the IV space (Martindale et al., 2008). Our current BOLD signal predictions, though extra-vascular, are sufficient at the current imaging resolution where we can only assume a mixed vascular region.

### Neuronal origin of negative BOLD

**Fig. 6:**
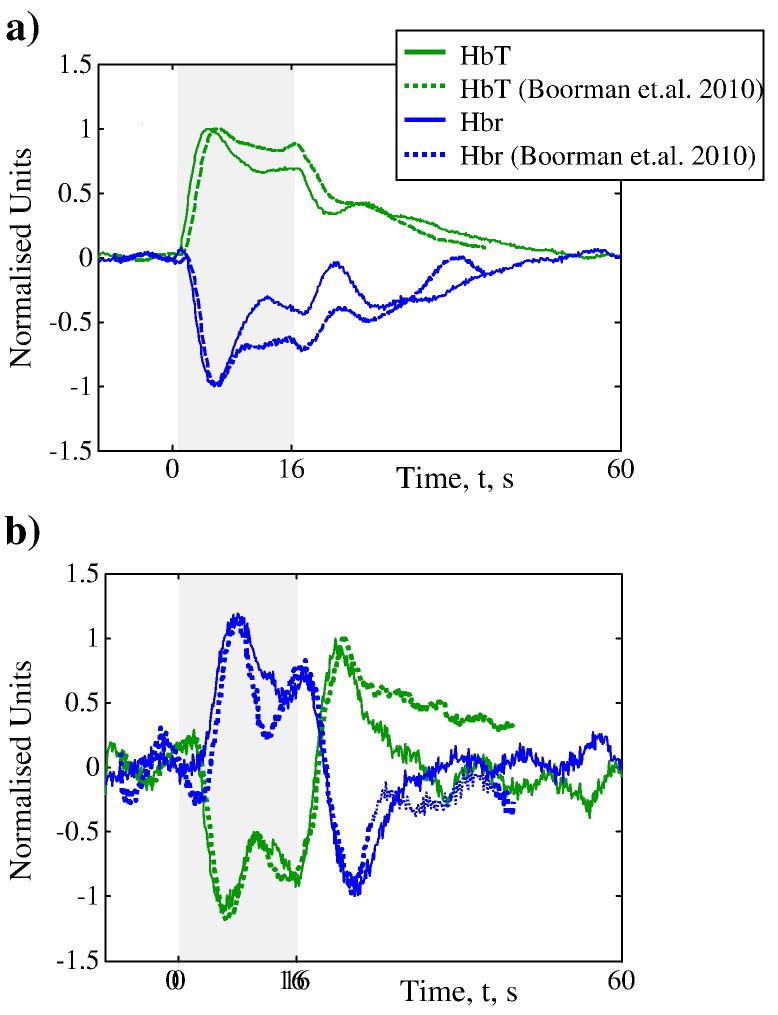
Direct comparison of haemodynamic time series (HbT and Hbr) from a) the whisker barrel region and b) the surrounding cortex to equivalent data from Boorman et al. (2010). Data has been normalised as Boorman et al. (2010) did not analyse the 2D-OIS data with improved tissue models. Furthermore, in that study *f*MRI and 2D-OIS were not performed concurrently so one could argue that the measured haemodynamics did not lead to a negative BOLD signal. We find that our normalised haemodynamic responses show similar transients to their 2D-OIS data. In the current study we have explicitly shown that such haemodynamic changes lead to a sustained negative BOLD signal, and thus our data adds more support to their hypothesis that the deep layer negative BOLD is driven by deep layer decreases in neuronal activity.

**Fig. 7:**
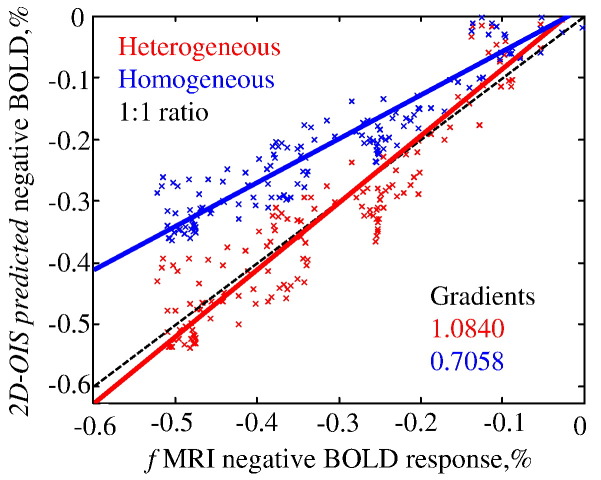
Correlation plot of predicted negative BOLD against measured fMRI negative BOLD. Negative data points from the mean times series across animals are plotted for both the 2D-OIS homogeneous (blue) and heterogeneous (red) tissue model time series predictions. For both tissue models there is a high correlation between prediction and measured BOLD (0.95). However, the gradient of heterogeneous tissue model (1.08) predictions overlap the 1:1 relationship, in contrast to the gradient of the homogeneous tissue model (0.71) which consistently under-estimates the magnitude of the BOLD response.

Boorman et al. (2010) hypothesised that the deep layer negative BOLD was driven by deep layer decreases in neuronal activity. However, in that study *f*MRI and 2D-OIS were not performed concurrently. In that study they did concurrent electrophysiology and 2D-OIS and compared data to coronal *f*MRI from a different set of subjects. We found that our normalised haemodynamic responses showed very similar transients to their 2D-OIS data (Fig. 6). As we have now explicitly shown that such haemodynamic changes lead to a negative BOLD signal, the current data adds more support to their proposal regarding the neuronal origin of the negative BOLD.

### Transients

Early increases in the concentration of Hbr at stimulus onset are thought to reflect the focal, capillary level, increases in CMRO_2_ (due to increased neuronal activity) before the larger surrounding blood vessels have dilated (Malonek and Grinvald, 1996, Mayhew et al., 2000). We observed an initial increase in Hbr levels in the whisker barrel cortical region following stimulus onset (Fig. 5b). A corresponding BOLD ‘initial dip’ was not observed in the *f*MRI time series (Fig. 5a). The absence of the BOLD initial dip is a direct consequence of low SNR and a 1 s TR, which is too long to capture this fast Hbr response. Future experiments with a shorter TR may more readily reveal the BOLD initial dip, but may also induce inflow effects into the *f*MRI data.

Under the assumption that the negative BOLD reflects decreases in neuronal activity, one may expect a decrease in Hbr levels in the surrounding cortical regions as CMRO_2_ decreases at stimulus onset. Interestingly, no biphasic behaviour in the Hbr response was observed in the surrounding cortical regions displaying negative BOLD signal changes.

Following stimulus offset and return to baseline conditions one may expect to observe a BOLD post-stimulus undershoot (Buxton et al., 1998, Dechent et al., 2011, Kruger et al., 1996, Mandeville et al., 1999, Yacoub et al., 2006, Zhao et al., 2007) The physiological source of the undershoot is attributed to elevated CMRO_2_ (Kruger et al., 1996), cerebral blood flow undershoots (Uludag et al., 2004) and/or delayed vessel compliance (Kong et al., 2004). Our data adds further to the conflicting evidence for this phenomenon, in the whisker barrel cortex we observe a post-stimulus ‘overshoot’ of ~ 1% BOLD in the whisker barrel cortex. The measured ‘overshoot’ is predicted from the underlying haemodynamics. We also observe the ‘overshoot’ in cortical areas which previously displayed a negative BOLD signal change during stimulation. Although this unexpected temporal dynamic could be a direct consequence of using Urethane anaesthetic in the rat model, it is nether-the-less interesting and requires further studies specifically aimed at the investigation of the source of this ‘overshoot’.

## Conclusion

We demonstrated the use of concurrent high field (7 T) *f*MRI and visible light 2D-OIS for the investigation of the haemodynamic response underlying the BOLD signal. We showed that despite the deep cortical origin of the negative BOLD response, if an appropriate heterogeneous tissue model is used in the spectroscopic analysis, 2D-OIS can be used to investigate this phenomenon. 2D-OIS data is recorded with high temporal and spatial resolution and offers a much cheaper option than *f*MRI for the investigation of the negative BOLD in the animal model. Furthermore it is more readily combined with electrophysiology (Harris et al., 2010; Kennerley et al., 2012) for investigations of neurovascular coupling.

This concurrent technology could be used for the investigation of other *f*MRI signal types. It would be possible to use this concurrent setup to measure (with *f*MRI) 3D cerebral blood volume or flow changes using VASO-MRI (Lu et al., 2003) and ASL (Wong et al., 1997) respectively and compare measurements in the cortex to the concurrent complementary haemodynamic as measured with 2D-OIS. This may be useful in helping parameterise these less established *f*MRI techniques for use in quantitative BOLD *f*MRI. The technology could also be important in any pre-clinical animal models of disease. Without the concurrent and complementary optical imaging measurements, abnormal BOLD signal changes could, at best, be difficult to interpret and, at worst, lead researchers to draw completely wrong conclusions from their data. BOLD *f*MRI imaging has revolutionised cognitive neuroscience research but now more than ever it needs complementary technologies to provide additional information to help understand what the BOLD signal changes mean especially in the context of disease where the less well understood negative BOLD signal may dominate.
